# Multiscale Interactome Analysis Prioritizes Candidate Herbs and Compounds for Diabetic Retinopathy

**DOI:** 10.3390/ijms27209066

**Published:** 2026-10-13

**Authors:** Yeong-Seon Ahn, Ji-In Chae, Seok-Hoon Han, Won-Yung Lee, Chihyun Park

**Affiliations:** 1 College of Korean Medicine, Wonkwang University, Iksan 54538, Republic of Korea; 2 Research Center of Traditional Korean Medicine, Wonkwang University, Iksan 54538, Republic of Korea; 3 Forensic Chemistry Division, National Forensic Service, 10 Ipchun-ro, Wonju-si 26460, Republic of Korea

**Keywords:** diabetic retinopathy, network pharmacology, multiscale interactome, biased random walk, herbal medicine, candidate compounds

## Abstract

Diabetic retinopathy (DR), a leading cause of preventable vision loss among working-age adults, arises from chronic hyperglycemic injury to the retinal microvasculature; current interventions such as anti-vascular endothelial growth factor (VEGF) injection are largely reactive, and mechanism-anchored herbal options remain undefined. We constructed a multiscale interactome and applied a biased random-walk diffusion model to prioritize herbal candidates, candidate compounds, and mechanisms for DR. Herb–ingredient records were drawn from OASIS; ingredient targets from DrugBank, TTD, and STITCH; and disease genes from DisGeNET. For each herb and compound, we computed diffusion profiles, correlated them with the DR profile, and assessed target-overlap significance by hypergeometric test. Eleven herbs combining high correlation (top 5%) with significant overlap (*p* < 0.05) were retained, led by Pini Koraiensis Semen (0.0263), Benincasae Exocarpium (0.0183), and Veratri Rhizoma Et Radix (0.0162); none had been previously investigated for DR. CASP3 emerged as a target shared by all eleven herbs, and enrichment analysis highlighted the advanced glycation end product–receptor for AGE (AGE–RAGE), TNF, IL-17, and NF-κB pathways alongside apoptosis and reactive-oxygen-species responses. Compound-level analysis recovered evidence-supported quercetin, resveratrol, oleic acid, luteolin, and diosgenin and nominated tectorigenin and cucurbitacins as novel candidates. This framework recapitulates known DR biology and elevates testable multi-target leads that warrant experimental validation for diabetic retinopathy.

## 1. Introduction

Diabetic retinopathy is a chronic microvascular complication of diabetes mellitus that arises from the prolonged exposure of the retinal vasculature to hyperglycemia [1]. The condition typically evolves from a non-proliferative stage, marked by microaneurysms, retinal hemorrhages, and increased vascular permeability, to a proliferative stage defined by pathological neovascularization, and it is frequently accompanied by diabetic macular edema [1]. Because these changes progressively damage the light-sensing retina, diabetic retinopathy has become a leading cause of preventable vision loss among working-age adults worldwide [2]. In 2020, an estimated 103 million adults were living with diabetic retinopathy, and this figure is projected to reach approximately 160 million by 2045 as the global prevalence of diabetes continues to rise [3]. This growing burden places considerable strain on health systems, largely because affected patients require long-term and repeated treatment [4]. At present, disease management depends on strict glycemic control combined with ophthalmic interventions such as laser photocoagulation, intravitreal anti-vascular endothelial growth factor (anti-VEGF) injection, and vitrectomy for advanced disease [5]. These options, however, are largely reactive rather than preventive, and they carry meaningful limitations, including the invasiveness and recurring cost of repeated injections, incomplete or transient responses in many patients, and procedure-related risks [5,6]. Safer and more mechanism-oriented therapeutic strategies are therefore still needed.

Herbal medicine, owing to its multi-component and multi-target nature, has attracted growing interest as a complementary strategy for chronic and multifactorial conditions such as diabetic retinopathy [7]. In traditional practice, diabetic retinopathy has been understood in terms of blood stasis and impaired ocular circulation, for which blood-activating herbs have long been prescribed [8]. Clinically, Compound Danshen Dripping Pills, a preparation combining *Salvia miltiorrhiza* (Danshen) and *Panax notoginseng* (Sanqi), have been assessed in randomized controlled trials and shown to improve visual acuity while reducing retinal hemorrhages and microaneurysms in patients with non-proliferative diabetic retinopathy [9]. A meta-analysis pooling 13 randomized controlled trials further corroborated these benefits, reporting improved overall clinical efficacy together with reductions in retinal microaneurysms and hemorrhages and a favorable safety profile in this setting [10]. Consistent with these clinical observations, preclinical studies have demonstrated that the same preparation protects retinal blood vessels and neurons in early diabetic retinopathy models, reflecting antioxidant, anti-inflammatory, and microvascular-protective actions [11]. Taken together, these clinical and experimental findings support the multi-target therapeutic potential of herbal medicine in diabetic retinopathy and motivate the systematic discovery of promising candidates with clarified mechanisms.

Despite these encouraging clinical and experimental data, the molecular mechanisms through which herbal medicines exert their effects in diabetic retinopathy remain incompletely defined, which hampers evidence-based translation into drug discovery [12]. Advances in systems biology have given rise to network pharmacology, an approach that dissects the complex relationships among herbal compounds, their protein targets, and disease-associated pathways at the systems level [13]. By representing these relationships as interconnected networks, this approach allows drug–target–disease connections to be visualized and the most influential mediators to be prioritized [14]. Network pharmacology has already proven valuable for uncovering the active compounds and mechanisms of herbal medicines across a range of chronic diseases [15]. However, conventional network pharmacology approaches typically infer herb–disease relevance from the direct overlap between compound targets and disease genes or from topological measures such as node degree within protein–protein interaction networks. These approaches have several shortcomings: they overlook herbs whose targets are not identical to disease genes but act on them indirectly through neighboring proteins, they tend to favor highly connected hub proteins, and they do not explicitly represent the biological functions through which target perturbations propagate to disease mechanisms. As a result, candidate rankings are often driven by the number of shared targets rather than by mechanistic proximity to the disease. More recently, the multiscale interactome—which models how perturbations propagate not only across protein–protein interactions but also through biological functions and pathways—was shown to predict the therapeutic effects of approved drugs more accurately than conventional network-based approaches [16]. This framework has since been extended to herbal medicine, efficiently prioritizing candidate herbs and their active compounds for hepatic cirrhosis and thereby demonstrating its utility for uncovering multi-target herbal mechanisms in complex diseases [17].

**Figure 1:**
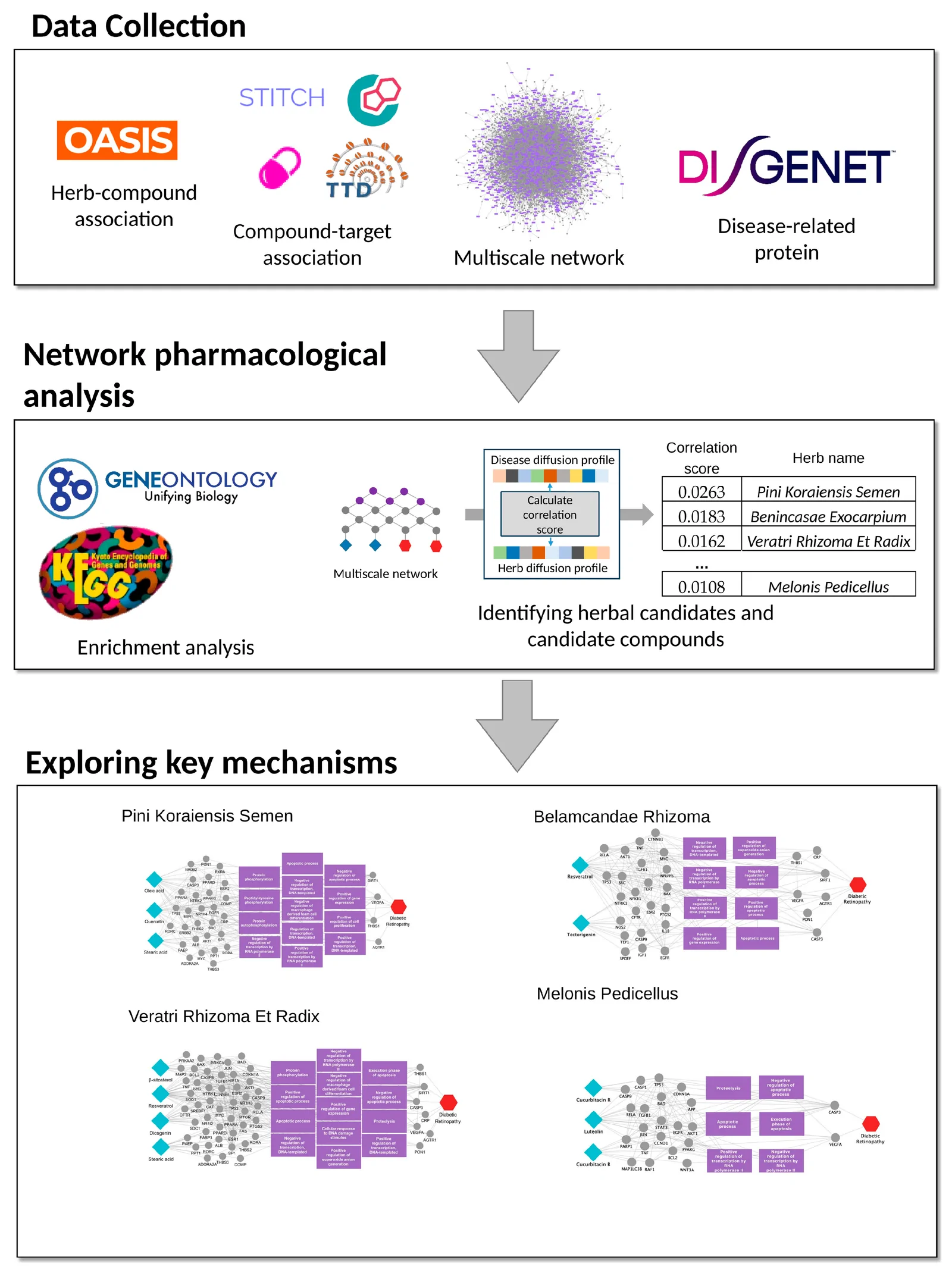
Overview of the study workflow. Herb–compound associations (OASIS), compound–target interactions (DrugBank, TTD, and STITCH), and diabetic retinopathy-associated proteins (DisGeNET) were integrated into a multiscale interactome. Diffusion profiles of herbs and compounds were correlated with the diabetic retinopathy profile to prioritize candidate herbs and candidate compounds, followed by functional enrichment analysis and exploration of the predicted mechanisms through compound–protein–biological function subnetworks.

In this study, we aimed to systematically identify herbal candidates and their constituent compounds potentially relevant to diabetic retinopathy and to elucidate their underlying molecular mechanisms through a multiscale interactome-based network pharmacology framework (Figure 1). We first assembled an integrated herb–compound–target knowledge base and curated a set of diabetic retinopathy-related disease targets. These data were then embedded into a multiscale network to derive diffusion profiles for the disease and for each candidate herb. By comparing herb-specific profiles with the diabetic retinopathy profile, we prioritized the herbs with the strongest disease relevance. We further characterized the key protein targets and enriched biological pathways associated with the top-ranked herbs. Finally, we extended the same analysis to the constituent compounds of these herbs in order to nominate disease-relevant candidate compounds and to infer their mechanistic roles. Collectively, this framework provides a principled route to prioritize multi-target herbal therapeutics for diabetic retinopathy and to link candidate herbs and compounds to disease-relevant mechanisms.

## 2. Results

### 2.1. Identification of Candidate Herbs for Diabetic Retinopathy

To prioritize herbs potentially relevant to diabetic retinopathy, we first assembled herb–ingredient relationships from the OASIS database and then retrieved the protein targets of these ingredients from validated resources, including DrugBank, TTD, and STITCH. Building on this herb–target information together with diabetic retinopathy-related targets and the proteins and biological functions embedded in the multiscale network, we implemented a biased random walk algorithm to derive diffusion profiles. For each herb, we subsequently computed a correlation score—reflecting how closely its diffusion profile resembled that of diabetic retinopathy—so that herbs achieving high scores could be prioritized as candidates for further investigation. In parallel, we applied a hypergeometric test to quantify the degree of protein overlap between each herb and the disease.

We prioritized herbs that ranked within the top 5% of all 349 herbs by correlation score, showed a statistically significant association (*p*-value < 0.05) with disease-related proteins, and contained at least three target-annotated compounds, which yielded eleven candidate herbs (Table S2). Among these, Pini Koraiensis Semen was the herb most strongly correlated with the diabetic retinopathy diffusion profile (correlation score 0.0263), followed by Benincasae Exocarpium (0.0183), Veratri Rhizoma Et Radix (0.0162), Maydis Stigma (0.0118), Pyrrosiae Folium (0.0116), Tribuli Fructus (0.0114), and Sparganii Rhizoma (0.0112); Smilacis Rhizoma, Belamcandae Rhizoma, Benincasae Semen, and Melonis Pedicellus followed closely, each with a score of 0.0108 when rounded to four decimal places (Table 1). The significant protein overlap and enrichment of these herbs (enrichment values ranging from 3.33 to 8.71) suggest that the model captured targets associated with the disease.

Because correlation scores are computed between diffusion profiles spanning approximately 29,000 nodes, their absolute values are inherently small and are best interpreted in relative terms. Across all 349 herbs, the median correlation score with the diabetic retinopathy profile was 0.0046 (interquartile range, 0.0043–0.0063), whereas the 11 candidate herbs scored 0.0108–0.0263, placing them within the top 5% of all herbs. For reference, ranibizumab, an anti-VEGF agent approved for diabetic retinopathy, and aflibercept showed correlation scores of 0.078 and 0.036, respectively, consistent with their direct targeting of VEGFA, a diabetic retinopathy-associated protein. In contrast, small-molecule agents used in or investigated for diabetic retinopathy, including fenofibrate, triamcinolone, dexamethasone, and epalrestat, showed scores of 0.0011–0.0038.

Notably, none of the eleven top-ranked herbs has substantial prior evidence directly supporting its use in diabetic retinopathy, which underscores the novelty of these network-based predictions. We therefore carried all eleven herbs forward for ingredient-level analysis and additionally selected four of them—Pini Koraiensis Semen, Veratri Rhizoma Et Radix, Belamcandae Rhizoma, and Melonis Pedicellus—which combined particularly high correlation scores with significant protein overlap, as representative candidates for detailed multiscale network visualization.

### 2.2. Herb–Ingredient–Target Network Construction of the Top-Ranked Herbs

**Figure 2:**
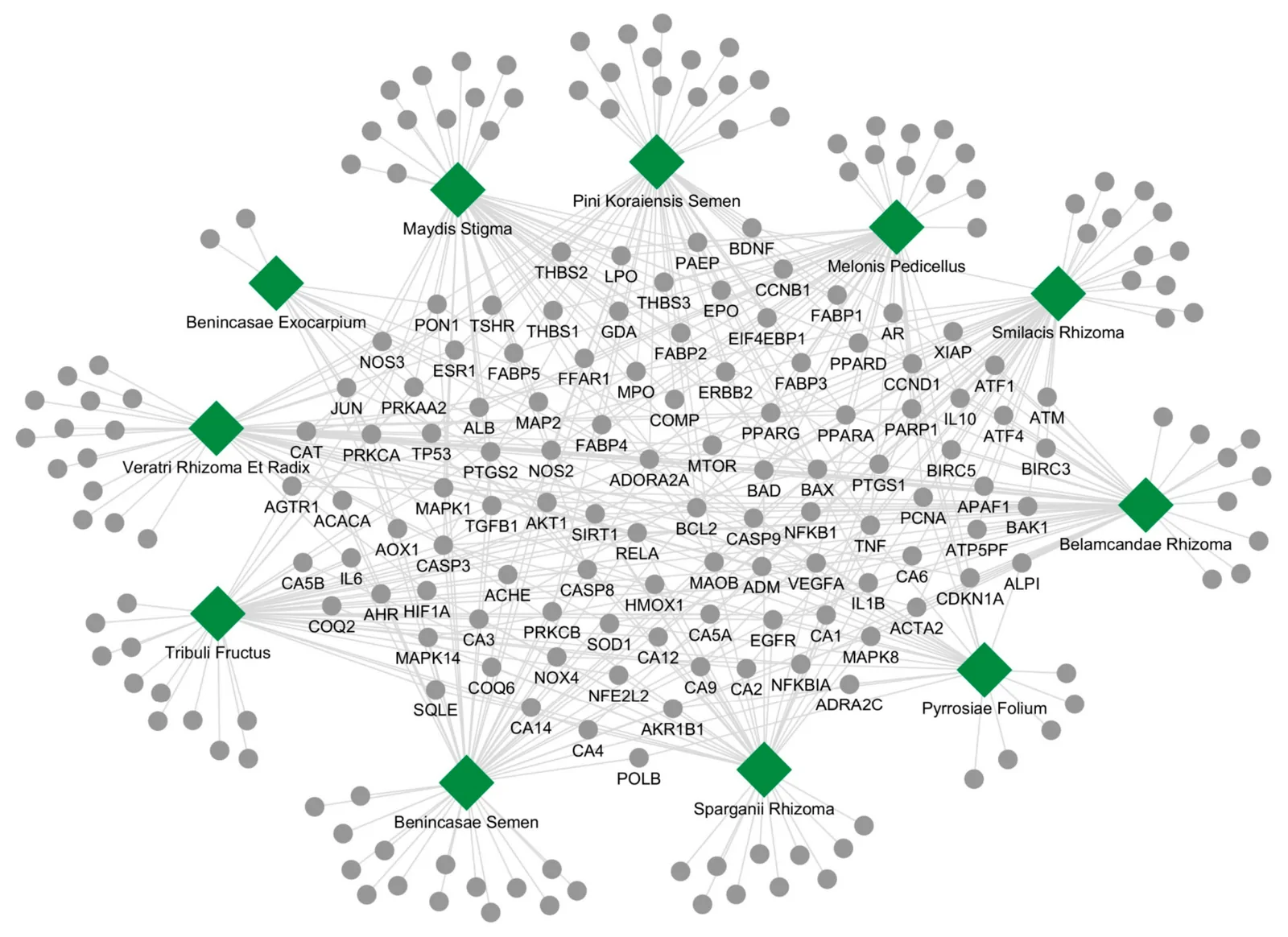
Herb–target interaction network of the 11 candidate herbs for diabetic retinopathy. Green diamonds denote herbs, and gray circles denote protein targets; all nodes are drawn at the same size. Edges represent herb–target interactions. The names of the 65 core protein targets—those shared by three or more of the 11 herbs—are labeled.

Next, using Cytoscape 3.10.2, we assembled and visualized an interaction network that maps the relationships between the eleven top-ranked herbs and their respective protein targets (Figure 2). The resulting network comprised 408 interactions (edges) linking 11 herbs to 156 targets, capturing the multi-component, multi-target nature of herbal medicine and the complex biological interactions through which their predicted effects may arise. Of these targets, three or more of the eleven herbs shared 65 proteins, marking them as core protein targets. CASP3 stood out as a target common to all eleven herbs, and the majority of the herbs each engaged PON1 and other proteins associated with inflammation and oxidative stress. To assess whether these associations were specific to diabetic retinopathy rather than driven by highly connected proteins, we performed two additional analyses. First, when the correlation scores of each candidate herb were ranked across all 840 diseases in the multiscale interactome, diabetic retinopathy ranked between 2nd and 18th for all 11 herbs (top 2.2%). Second, after removing CASP3, TNF, IL6, and IL1B from both the herb targets and the background, the target overlap with the diabetic retinopathy protein set remained significant for all 11 herbs (*p* = 1.4 × 10^−7^ to 0.028), indicating that the overlap was not attributable solely to these widely shared inflammatory and apoptotic proteins. Collectively, these observations suggest that the core protein targets may contribute to the predicted mechanisms of these herbs in diabetic retinopathy.

### 2.3. Functional Enrichment Analysis of the Top-Ranked Herbs

**Figure 3:**
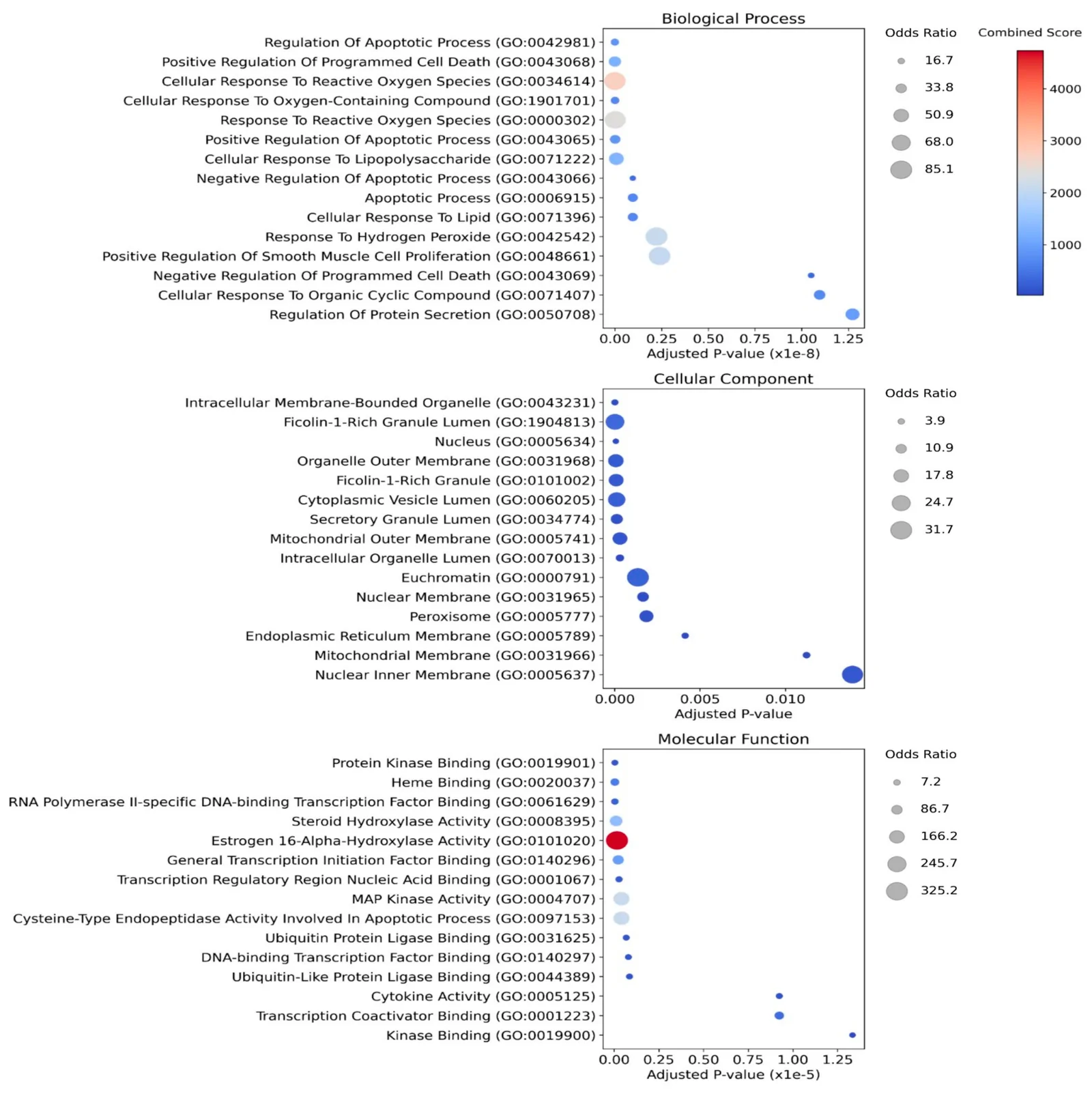
Gene Ontology enrichment analysis of the 65 core protein targets across three categories: biological processes (**top**), cellular components (**middle**), and molecular functions (**bottom**). The x-axis shows the adjusted *p*-value (association significance), bubble size corresponds to the odds ratio, and bubble color reflects the combined score, which indicates the statistical significance of each term. The most significantly enriched terms in each category are highlighted.

To characterize the biological relevance of these core protein targets, we performed functional enrichment (over-representation) analysis against the KEGG and Gene Ontology databases, identifying the signaling pathways and biological functions associated with the selected top-ranked herbs. The KEGG analysis linked the targets to several pathologically relevant pathways, most notably the AGE–RAGE signaling pathway in diabetic complications, the C-type lectin receptor signaling pathway, and pathways of neurodegeneration; inflammatory pathways such as the TNF and IL-17 signaling pathways, together with lipid and atherosclerosis, likewise ranked among the leading enriched terms (Table 2). Gene Ontology analysis revealed strong associations with essential biological functions, chiefly those governing the regulation of apoptosis and the response to reactive oxygen species, while inflammation-related cellular responses to lipopolysaccharide and lipids were also significantly represented (Figure 3, top). Examining cellular components, the core targets localized predominantly to the mitochondria, nucleus, cytoplasmic vesicles, secretory granules, and peroxisomes—organelles essential for cell survival, signal transduction, and retinal cell homeostasis (Figure 3, middle). In the molecular function category, activities related to oxidative stress regulation and cytokine signaling showed high combined scores, indicating strong associations (Figure 3, bottom). Taken together, these results suggest that the core protein targets are associated with the signaling pathways and biological functions underlying diabetic retinopathy and provide a basis for further experimental investigation.

### 2.4. Prioritization of Candidate Compounds and Multiscale Network Mechanism Analysis of the Candidate Herbs

We then examined the ingredients and multiscale network mechanisms of the eleven top-ranked herbs to predict how their constituents might act on diabetic retinopathy. For each compound, we fed target information into the multiscale network analysis to compute a correlation score reflecting its predicted association with the disease. Compounds with significant target overlap (*p* < 0.05) were designated as prioritized candidate compounds (Table 3); this term denotes computationally prioritized constituents and does not imply experimentally demonstrated activity.

Across the eleven herbs, our analysis prioritized a mix of shared and herb-specific candidate compounds. In Pini Koraiensis Semen, oleic acid (0.0176), quercetin (0.0150), and stearic acid (0.0108) emerged as the leading constituents. Our analysis recurrently prioritized β-sitosterol (0.0243), which ranked as the top-scoring ingredient in Benincasae Exocarpium, Maydis Stigma, Pyrrosiae Folium, Tribuli Fructus, and Benincasae Semen. Veratri Rhizoma Et Radix was characterized by β-sitosterol (0.0243), resveratrol (0.0170), diosgenin (0.0113), and stearic acid (0.0108); Maydis Stigma additionally by oleic acid (0.0176), palmitic acid (0.0070), and piperine (0.0054); Tribuli Fructus by dioscin (0.0089); and Sparganii Rhizoma by hydroxytyrosol (0.0186). Resveratrol (0.0170) defined Smilacis Rhizoma and, together with tectorigenin (0.0097), Belamcandae Rhizoma, while Benincasae Semen further featured quercetin (0.0150) and rutin (0.0085). For Melonis Pedicellus, we identified cucurbitacin R (0.0192), oleic acid (0.0176), cucurbitacin B (0.0116), stearic acid (0.0108), palmitic acid (0.0070), and luteolin (0.0050) as candidate compounds—each showing a high correlation score and statistically significant protein overlap within the diabetic retinopathy diffusion profile (Table 3).

Many of the prioritized ingredients—such as quercetin [18,19], resveratrol [20,21,22,23], oleic acid [24], luteolin [25], diosgenin [26,27], hydroxytyrosol [28], and rutin [29]—already have documented therapeutic or protective effects relevant to diabetic retinopathy or its underlying pathology. By contrast, tectorigenin from Belamcandae Rhizoma and cucurbitacin R and cucurbitacin B from Melonis Pedicellus have little or no prior evidence of efficacy for this condition, suggesting that they may represent novel candidate compounds for further investigation. This combination of established and previously unreported constituents suggests that our approach prioritized both previously reported and previously unexplored compounds associated with diabetic retinopathy.

**Figure 4:**
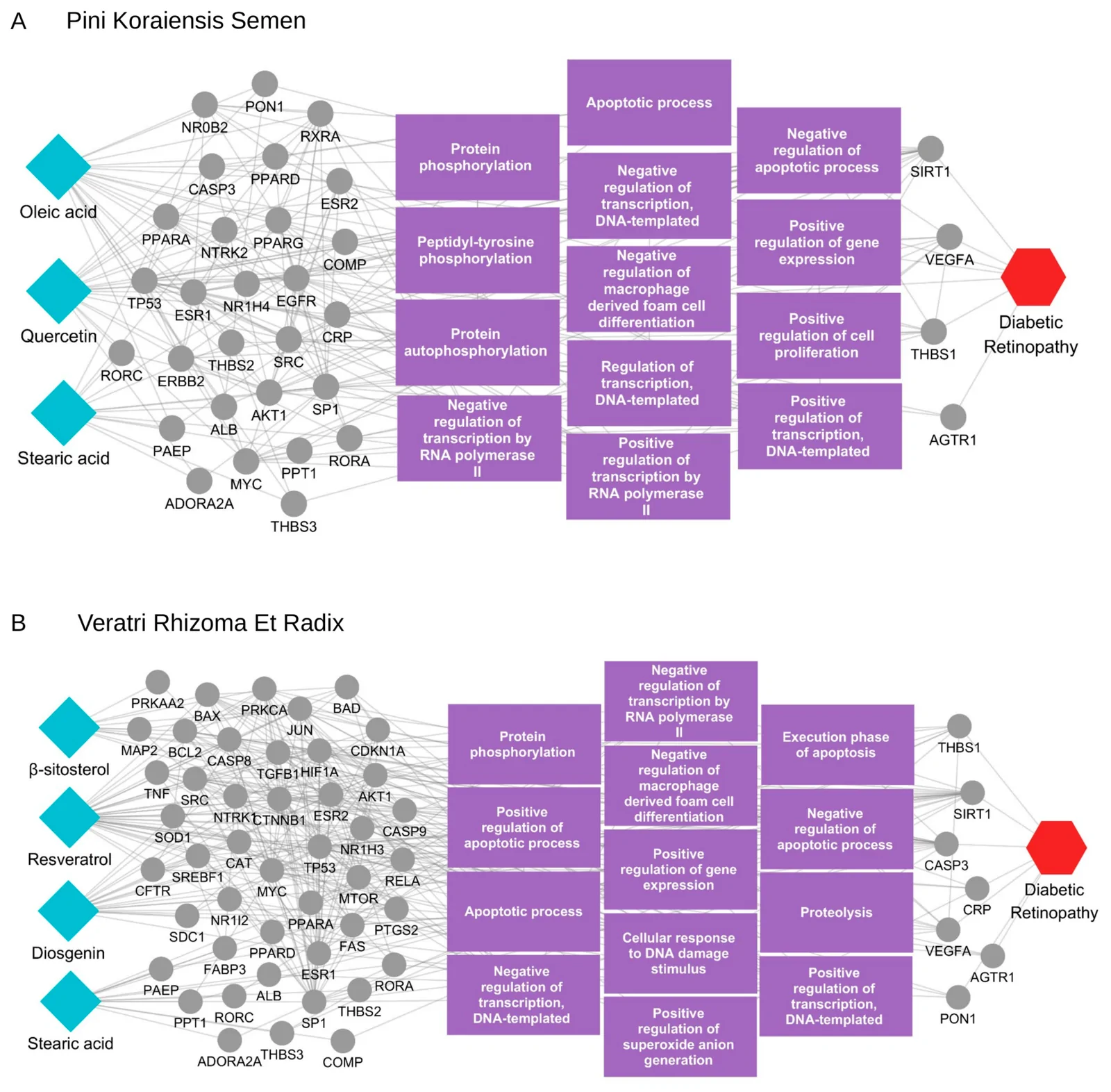
Predicted multiscale network mechanisms of candidate compounds from the candidate herbs. (**A**) Oleic acid, quercetin, and stearic acid from Pini Koraiensis Semen; (**B**) β-sitosterol, resveratrol, diosgenin, and stearic acid from Veratri Rhizoma Et Radix. Light-blue diamonds indicate the candidate compounds, gray circles the protein targets, and the red hexagon the disease of interest. Purple rectangles highlight the distinct biological functions associated with the targets.

To probe the key mechanisms behind these candidate compounds, we constructed subnetworks that link each ingredient to the protein targets and biological functions most strongly affected in diabetic retinopathy, focusing the detailed visualization on the four representative herbs. For Pini Koraiensis Semen, oleic acid, quercetin, and stearic acid were predicted to interact directly with apoptosis-, growth factor signaling-, and nuclear receptor-related proteins, including CASP3, TP53, AKT1, EGFR, PPARA, and PPARG, whereas other proteins participated in disease-associated functions or were indirectly connected to the disease proteins. The functions represented—regulation of transcription and gene expression, protein phosphorylation, and apoptosis—are all implicated in diabetic retinopathy (Figure 4A). A comparable pattern was observed for Veratri Rhizoma Et Radix, in which β-sitosterol, resveratrol, diosgenin, and stearic acid were predicted to interact with TNF, CASP3, CASP9, BAX, and CAT and to be linked, directly or indirectly, to processes such as superoxide anion generation, the cellular response to DNA damage, regulation of gene expression, and the apoptotic process (Figure 4B).

**Figure 5:**
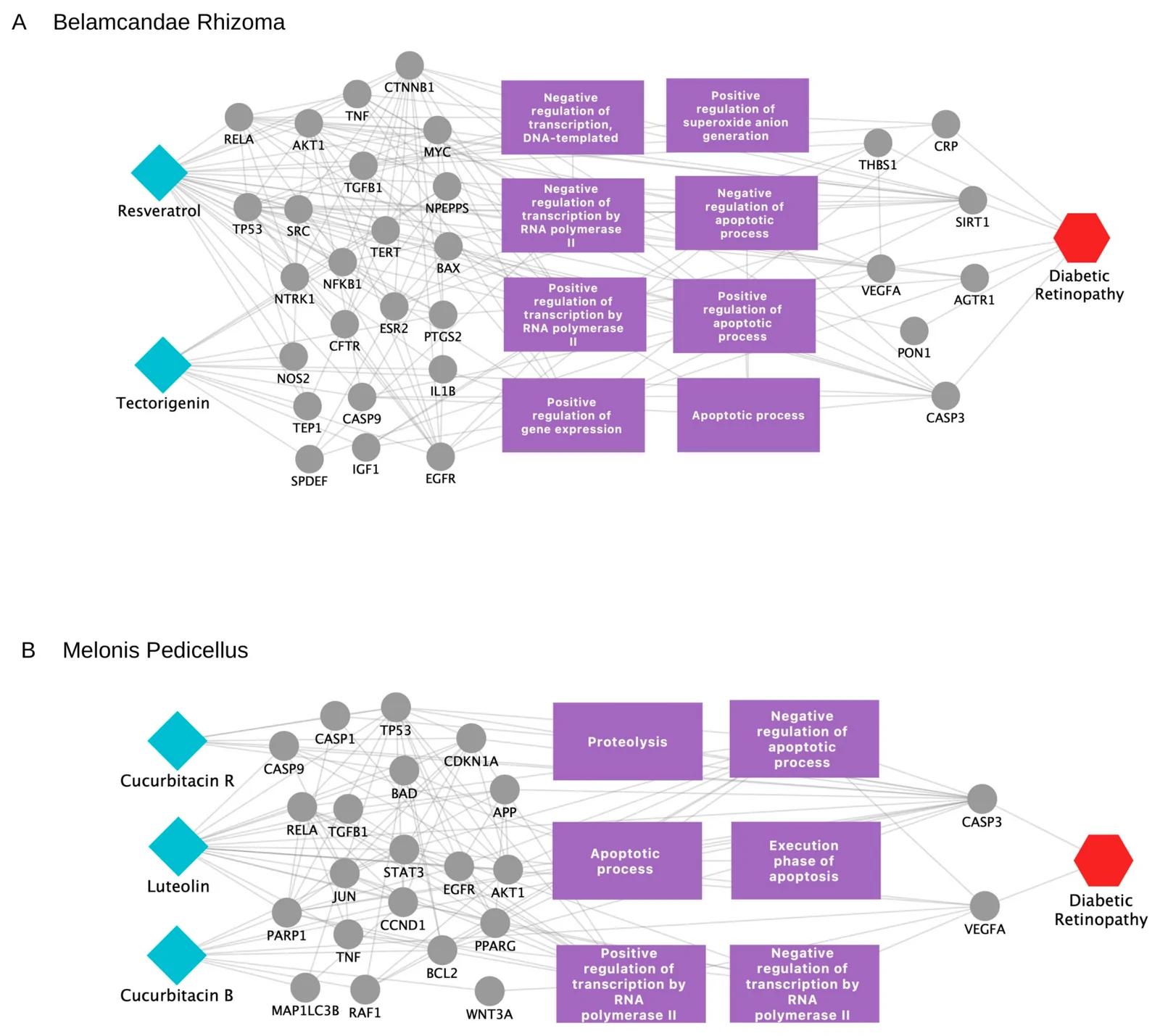
Predicted multiscale network mechanisms of candidate compounds from candidate herbs lacking prior evidence. (**A**) Resveratrol and tectorigenin from Belamcandae Rhizoma; (**B**) cucurbitacin R, cucurbitacin B, and luteolin from Melonis Pedicellus. Light-blue diamonds indicate the candidate compounds, gray circles the protein targets, and the red hexagon the disease of interest. Purple rectangles highlight the distinct biological functions associated with the targets.

We next investigated the herbs whose candidate compounds had previously lacked evidence, mapping the predicted molecular interactions that might underlie their potential effects. The network indicated that tectorigenin and resveratrol from Belamcandae Rhizoma were predicted to act directly on inflammation- and apoptosis-related proteins including IL1B, TNF, CASP3, and CASP9, and to be linked to other proteins networked with disease-linked targets that govern gene expression, transcriptional activity, and core cellular processes such as apoptosis and superoxide anion generation (Figure 5A). Finally, we visualized the predicted associations of the Melonis Pedicellus ingredients cucurbitacin R, cucurbitacin B, and luteolin (Figure 5B): these compounds were predicted to interact directly with inflammation- and apoptosis-related proteins such as TNF, CASP3, CASP9, BAD, and BCL2, and to be linked to disease-associated biological functions and, indirectly, to diabetic retinopathy-associated proteins. Their predicted associations again converged on transcriptional regulation and apoptosis. In summary, these findings nominate candidate compounds from the candidate herbs for further investigation and provide mechanistic hypotheses by predicting the biological functions of the proteins that may mediate their associations with diabetic retinopathy.

### 2.5. Molecular Docking and Molecular Dynamics Analysis

**Figure 6:**
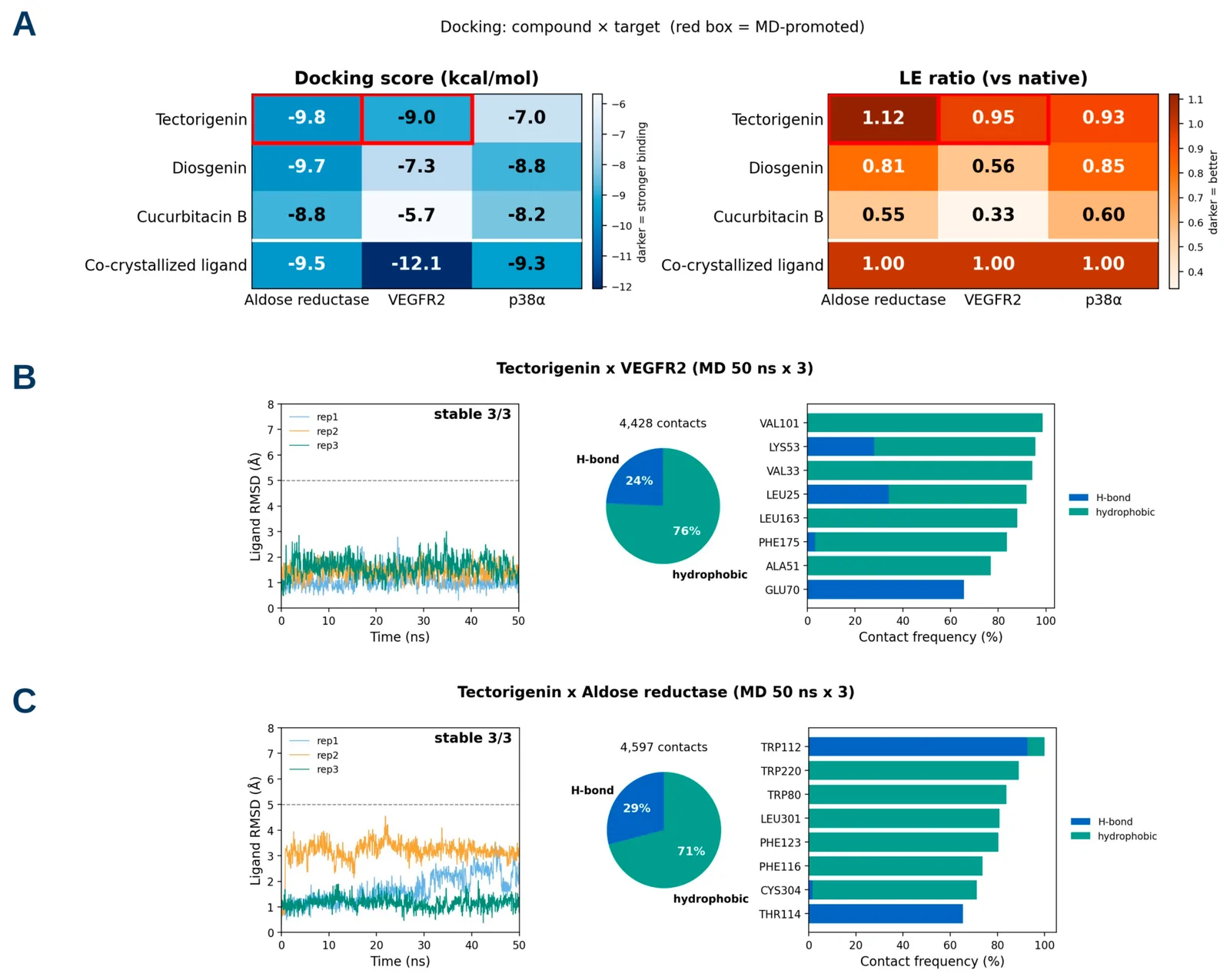
Docking and molecular dynamics analysis of candidate compounds. (**A**) AutoDock Vina docking scores (kcal/mol) and ligand-efficiency (LE) ratios of three candidate compounds (tectorigenin, diosgenin, and cucurbitacin B) and the co-crystallized ligands against aldose reductase (PDB ID 1US0), VEGFR2 (PDB ID 4AG8), and p38α (PDB ID 1A9U); LE ratios are expressed relative to the co-crystallized ligand of each protein. (**B**,**C**) Three independent 50-ns MD simulations (replicates shown separately) of the tectorigenin–VEGFR2 (**B**) and tectorigenin–aldose reductase (**C**) complexes, showing ligand RMSD, interaction-type distribution, and residue contact frequencies. The dashed line indicates the 5 Å threshold used to classify a replicate as stable.

Three candidate compounds were docked against aldose reductase, VEGFR2, and p38α: tectorigenin and cucurbitacin B, which lacked prior evidence for diabetic retinopathy, and diosgenin, an evidence-supported comparator (see Section 4.5 for the selection criteria). Among them, tectorigenin showed the most favorable overall docking profile, with docking scores of −9.8, −9.0, and −7.0 kcal/mol and LE ratios of 1.12, 0.95, and 0.93 against aldose reductase, VEGFR2, and p38α, respectively (Figure 6A). Relative to the co-crystallized inhibitors, the LE ratio of tectorigenin exceeded 1.0 for aldose reductase and approached 1.0 for VEGFR2 and p38α, whereas diosgenin and cucurbitacin B showed lower ligand efficiencies (Table S6). Triplicate MD simulations further suggested stable binding of tectorigenin to both VEGFR2 and aldose reductase, with mean last-half ligand RMSDs of 1.39 ± 0.25 Å and 2.13 ± 0.85 Å, respectively (Figure 6B,C). Under the same conditions, the co-crystallized ligands axitinib and IDD594 showed last-half RMSDs of 0.91 ± 0.17 Å and 1.79 ± 0.62 Å, respectively; thus, the tectorigenin poses were slightly less stable than, but broadly comparable to, those of the known inhibitors (Table S6). Contact analysis showed persistent interactions within the VEGFR2 ATP-binding pocket and the aromatic binding pocket of aldose reductase, supporting the structural plausibility of tectorigenin as a prioritized candidate; unlike IDD594, however, tectorigenin did not occupy the catalytic anion-binding site of aldose reductase. However, these docking and MD results indicate only the structural compatibility of tectorigenin with the binding sites of these proteins and do not demonstrate target inhibition or therapeutic activity, which must be confirmed experimentally. In silico ADMET prediction indicated that tectorigenin satisfied Lipinski’s rule of five and was predicted to have high gastrointestinal absorption, whereas cucurbitacin B was predicted to have low gastrointestinal absorption, consistent with its reported low oral bioavailability [30] (Table S7).

## 3. Discussion

This study applied a multiscale interactome with a biased random-walk algorithm to nominate candidate herbs and compounds for diabetic retinopathy and to investigate their putative mechanisms of action. Among the top-ranked herbs that showed high correlation scores with the disease diffusion profile and statistically significant target overlap (*p*-value < 0.05), we identified Pini Koraiensis Semen, Benincasae Exocarpium, Veratri Rhizoma Et Radix, Maydis Stigma, Pyrrosiae Folium, Tribuli Fructus, Sparganii Rhizoma, Smilacis Rhizoma, Belamcandae Rhizoma, Benincasae Semen, and Melonis Pedicellus. None of these herbs have been directly investigated for diabetic retinopathy, so all eleven emerge here as novel candidates. These findings deepen our understanding of how multi-component traditional herbs may act on diabetic retinopathy and provide testable hypotheses for future experimental studies.

The multiscale network approach allowed us to evaluate broad herb–disease relationships across the human interactome, extending beyond direct protein–protein interactions to incorporate biological functions and pathways [16]. Prior work suggests that this strategy can outperform alternative network-based methods in recovering disease-relevant herbal compounds and their mechanisms [31]. By simulating diffusion with a biased random walk and computing interaction similarity, we improved predictive resolution because the algorithm shifts transition probabilities toward biological-function nodes rather than relying on protein nodes alone. This framework provided insights that are not available from target-overlap analysis alone. First, the ranking by diffusion-profile correlation differed from the ranking by overlap significance; for example, Pini Koraiensis Semen ranked higher by correlation than Veratri Rhizoma Et Radix despite sharing fewer disease proteins (3/50 vs. 5/50), indicating that the model captures mechanistic proximity beyond the number of shared targets. Second, the compound-level subnetworks identified the intermediate proteins and biological functions, such as apoptotic regulation, transcriptional regulation, and superoxide anion generation, through which compound targets were predicted to connect to diabetic retinopathy-associated proteins, thereby providing testable mechanistic hypotheses rather than lists of shared targets. Third, the correlation scores enabled disease-specific comparison across all 840 diseases in the interactome, which showed that the candidate herbs were preferentially associated with diabetic retinopathy. This design let us capture how herb and ingredient targets influence the core pathways and mechanisms of diabetic retinopathy at a systems level. Having previously been applied to nominate polyphenols for oxidative liver injury [32], the framework was here extended to diabetic retinopathy, where it prioritized candidate herbs and core protein targets that impinge on disease-salient pathways.

Enrichment analysis of the core targets delineated the signaling pathways and biological functions through which the prioritized herbs may act. The KEGG results highlighted the AGE–RAGE signaling pathway in diabetic complications, together with inflammatory axes such as the TNF and IL-17 signaling pathways and pathways of neurodegeneration—routes that are central to the microvascular and neuronal damage of diabetic retinopathy [33]. Gene Ontology analysis converged on the regulation of apoptosis, the response to reactive oxygen species, and inflammatory responses, while the core targets localized to organelles such as the mitochondria, nucleus, and peroxisomes that govern cell survival and redox control. Because chronic hyperglycemia drives oxidative stress, vascular inflammation, and progressive retinal apoptosis [5], these results suggest that the prioritized herbs and their constituent compounds may be associated with the intertwined pathways of inflammation, oxidative stress, and cell death.

At the ingredient level, our analysis nominated both well-supported and previously under-recognized candidate compounds. Compounds such as quercetin, resveratrol, oleic acid, luteolin, diosgenin, hydroxytyrosol, and rutin already have documented protective effects relevant to diabetic retinopathy or its underlying pathology, which is consistent with the network-based prioritization. In contrast, tectorigenin from Belamcandae Rhizoma and cucurbitacin R and cucurbitacin B from Melonis Pedicellus have little or no prior evidence for this condition, so they emerge as novel mechanistic hypotheses. Molecular docking and MD simulations further suggested that tectorigenin can stably occupy the binding sites of aldose reductase and VEGFR2; however, these structural predictions do not establish inhibitory or therapeutic activity, and enzymatic assays and cell-based studies under high-glucose conditions are required to test these hypotheses. The compound-centric subnetworks provided further resolution, as the prioritized compounds were predicted to interact with pro-inflammatory and apoptosis-related proteins such as TNF, IL1B, CASP3, CASP9, and CAT and converged on the regulation of gene expression, apoptosis, and superoxide anion generation. Unlike earlier herbal studies that examined single herbs or isolated compounds, our framework links candidate herbs and their constituents to disease-relevant proteins and functions within one integrated model, thereby offering testable mechanistic hypotheses for future validation.

The translational interpretation of these findings should also consider the safety of the prioritized herbs. A high network correlation score reflects predicted proximity to disease mechanisms and does not indicate that an herb is suitable for therapeutic use. Several candidates carry known toxicity concerns. Veratri Rhizoma Et Radix contains steroidal alkaloids that act on neuronal sodium channels and can cause vomiting, bradycardia, hypotension, and cardiac conduction abnormalities; *Veratrum* preparations were historically withdrawn as antihypertensive agents because of their narrow therapeutic index [34]. Likewise, cucurbitacins, the major constituents of Melonis Pedicellus, show non-specific cytotoxicity and low oral bioavailability [30], so their predicted interactions may not translate into a favorable efficacy–safety balance in vivo. For these herbs, dose, route of administration, pharmacokinetics (including penetration of the blood–retinal barrier), and the therapeutic window would need to be established before any clinical application can be considered. Accordingly, the herbs and compounds prioritized here should be regarded as candidates for further pharmacological and toxicological evaluation rather than as agents ready for therapeutic use.

Several limitations should be considered when interpreting these findings. First, the analysis relies on public databases that are inherently incomplete, and many constituents and interactions remain unrecorded. Second, these databases are subject to literature bias, because well-studied compounds and proteins accumulate more annotations and are more likely to be prioritized. Third, network diffusion favors highly connected hub proteins; although the target overlap was robust to the removal of CASP3, TNF, IL6, and IL1B, the correlation scores were not recomputed after hub removal. Fourth, the multiscale interactome is not tissue-specific, so some predicted interactions may not occur in retinal cells. Fifth, herbal composition is uncertain: herb-level targets were inferred from documented constituents without weighting by their abundance, and whole herbs, extracts, and isolated compounds are not pharmacologically interchangeable. In addition, the disease proteins were drawn from a single curated resource, and pharmacokinetic properties were considered only through in silico ADMET predictions for the docked compounds (Table S7). The MD simulations were limited to three 50-ns replicates, which may not capture slower conformational changes, and the aldose reductase simulations did not include the NADP^+^ cofactor. Finally, the biased random-walk algorithm does not specify whether a compound activates or inhibits its targets; because activation of CASP3 or pro-inflammatory cytokines such as TNF, IL6, and IL1B could aggravate rather than alleviate retinal injury, the predicted associations should not be interpreted as beneficial effects, and experimental studies are required to determine their direction.

## 4. Materials and Methods

### 4.1. Herb–Ingredient–Target Network Construction

Data on herbs and their constituent ingredients were obtained from the OASIS traditional medicine database (https://oasis.kiom.re.kr/index.jsp; accessed on 7 March 2026), which is maintained by the Korea Institute of Oriental Medicine (KIOM). Within this platform, constituents isolated from herbs are catalogued on the basis of physicochemical profiling methods—including HPLC and UPLC—and are validated by pharmacological and traditional medicine experts. For the present work, 13,491 herb–ingredient records covering 420 herbs were retrieved from OASIS. Records without a PubChem CID were removed, compounds were standardized by PubChem CID, and duplicate herb–compound pairs were merged, yielding 7552 unique herb–compound associations spanning 364 herbs and 4786 compounds. Of these, 349 herbs contained at least one compound with target information and were included in the network analysis. These data were used as the foundational input for the subsequent network analysis. Herbal materials are referred to by their pharmacopoeial Latin names; their botanical origins and medicinal parts were verified against the Korean Pharmacopoeia and the Korean Herbal Pharmacopoeia using the KIOM Defining Dictionary for Medicinal Herbs (https://oasis.kiom.re.kr/herblib/hminfo/hbmcod/hbmcodList.do; accessed on 28 September 2026), and currently accepted botanical names and families were confirmed in Plants of the World Online (https://powo.science.kew.org/; accessed on 28 September 2026) (Table 1). Ingredient–target interactions supported by experimental evidence were then compiled from authoritative databases, including DrugBank 5.0 [35], the Therapeutic Target Database (TTD) [36], and the Search Tool for Interactions of Chemicals (STITCH 5) [37], which provide extensive information on established and potential targets, associated diseases, biological pathways, and the drugs that act on these proteins. Only interactions supported by experimental evidence were retained; for STITCH, associations derived solely from text mining or computational prediction were excluded.

A network was subsequently constructed to represent the relationships among herbs, ingredients, and protein targets. Ingredients identified through PubChem CID were cross-referenced and integrated with the ingredient–target data, and herbs with fewer than three target-associated ingredients were not considered as candidate herbs so that the reliability of the predictions could be strengthened, although their scores are reported in Table S2 for completeness. Interactions among herbs, components, and protein targets were then visualized and analyzed through the resulting network. In this framework, the targets of an herb were defined as the union of the protein targets of its constituent compounds recorded in OASIS. To reduce the influence of targets supported by only a single constituent, targets were ranked by path count, that is, the number of constituent compounds in the herb that interact with each target, and up to 50 top-ranked targets were retained per herb. This representation reflects the predicted target space of the documented constituents and does not account for their relative abundance in the herb. Accordingly, throughout this study, “herb” refers to a computational representation of a medicinal material defined by its documented constituents and their targets, rather than to a specific extract or preparation, and “compound” refers to an individual constituent analyzed as a single chemical entity. The pharmacological properties of whole herbs, extracts, and isolated compounds are not interchangeable, and our predictions do not directly apply to any particular extract. Database versions and access dates are summarized in Table S5.

### 4.2. Functional Enrichment Analysis

Biological processes and signaling pathways associated with the 65 core protein targets were analyzed by functional enrichment analysis based on over-representation analysis (ORA), which was performed through the Enrichr platform (http://amp.pharm.mssm.edu/Enrichr/; accessed on 29 March 2026) using the enrichr function of the GSEApy module (version 1.1.3) [38] in a Python 3.7 environment. The KEGG_2021_Human, GO_Biological_Process_2023, GO_Cellular_Component_2023, and GO_Molecular_Function_2023 libraries were used (accessed on 29 March 2026). Diverse gene-set libraries, including Gene Ontology [39] and the Kyoto Encyclopedia of Genes and Genomes (KEGG) [40], are drawn upon by Enrichr to perform the enrichment analysis. Adjusted *p*-values, z-scores, and combined scores were calculated to evaluate the signaling pathways and biological functions related to the targets of herbal ingredients. The combined score, which is derived by multiplying the logarithm of the *p*-value by the z-score, was used to rank the enriched pathways and biological functions associated with the core targets. All signaling pathways identified through the enrichment analysis were retained, except for those tied specifically to diseases.

### 4.3. Disease-Related Targets

Diabetic retinopathy-related protein data were curated following the approach of Ruiz et al. [16] and were obtained from DisGeNET (https://www.disgenet.org/; accessed on 7 March 2026), a database in which disease–gene associations are meticulously mapped to ensure high reliability [41]. Only expert-curated disease–gene associations provided by DisGeNET for “Diabetic Retinopathy” (UMLS Concept Unique Identifier: C0011884) were considered, so that relevance to the study could be maintained. Data in this curated set were drawn from reputable sources, including UniProt, the Comparative Toxicogenomics Database, Orphanet, the Clinical Genome Resource, the Genomics England PanelApp, the Cancer Genome Interpreter, and the Psychiatric Disorders Gene Association Network. Disease–gene associations based on homology in animal models or derived from computational literature mining, as well as those labeled as therapeutic, were excluded. The refined disease–protein interaction network served as the basis for validating diabetic retinopathy-related protein data in this study.

### 4.4. Multiscale Network Analysis for Predicting Disease Associations

A multiscale interactome was constructed using the data and methodology of Ruiz et al. [16], in which three types of interactions were integrated: protein–protein, protein–biological function, and biological function–function interactions. Human protein–protein interaction data were obtained from sources such as the Biological General Repository for Interaction Datasets (BioGRID), the Database of Interacting Proteins (DIP), and the Human Reference Protein Interactome Mapping Project (HuRI), encompassing 387,626 physical interactions among 17,660 proteins. Protein–biological function interactions were extracted from the human Gene Ontology database, encompassing 34,777 experimentally verified associations between 7993 proteins and 6387 biological functions. Biological function–function interactions were organized into a hierarchical network with 22,545 associations among 9798 functions. Diffusion profiles were then computed on the multiscale interactome so that the propagation effects of herbal ingredients on diabetic retinopathy-associated proteins could be evaluated. In this analysis, a biased random walk with restart algorithm was applied to quantitatively assess the influence exerted by herbal targets and ingredient targets on diabetic retinopathy-related proteins. Correlation scores between the herb/ingredient profiles and the disease profile were subsequently calculated, by which candidate herbs and ingredients potentially relevant to diabetic retinopathy could be prioritized.

Diffusion profiles were computed using the optimized hyperparameters reported by Ruiz et al. [16] (α = 0.8595; maximum of 1000 iterations; convergence tolerance of 10^−6^; node-type edge weights as listed in Table S5), and the correlation score was calculated as one minus the uncentered correlation (cosine) distance between two diffusion profiles, so that higher scores indicate more similar propagation patterns. For candidate selection, herbs were ranked by correlation score, and a high correlation score was defined as ranking within the top 5% of all 349 herbs; herbs within this range that also showed significant target overlap (*p* < 0.05) and contained at least three target-annotated compounds were selected as candidate herbs, and their constituent compounds with significant target overlap (*p* < 0.05) were designated as candidate compounds. Overlap significance was assessed with a one-sided hypergeometric test. The background set was defined as the union of all drug-side target proteins and all disease-associated proteins in the multiscale interactome: 5918 proteins for the herb-level analysis (1357 herb targets and 5608 disease proteins) and 8285 proteins for the compound-level analysis (5575 compound targets and 5608 disease proteins). The diabetic retinopathy gene set comprised eight proteins (CASP3, GAD2, VEGFA, SIRT1, THBS1, AGTR1, PON1, and CRP). *p*-values were adjusted for multiple testing using the Benjamini–Hochberg false discovery rate procedure, and adjusted values (q-values) are reported alongside nominal *p*-values in Table 1 and Table 3.

To identify the principal mechanisms of each ingredient–disease pair, the diffusion profiles were examined and the top k proteins or biological functions—ranked according to their influence from either the drug or the disease side—were extracted. A network was then constructed from these entities so that their relevance could be highlighted, and ingredient targets that showed no connection to disease-related proteins or biological functions were excluded. The entity ranked highest in the diffusion profile was regarded as the most influential in the predicted mechanism, owing to its pronounced influence. A value of k = 20 was chosen so that a substantial share of the visitation frequency within the diffusion profile could be captured. Complete details of the diffusion profile computation—covering the mathematical formulation, the iterative scheme, and the rationale behind the choice of k—are available in prior research [16].

### 4.5. Molecular Docking, Molecular Dynamics Simulations, and ADMET Prediction

Compounds and target proteins for docking were selected as follows. Tectorigenin and cucurbitacin B were chosen as candidate compounds lacking prior evidence for diabetic retinopathy (Table 3); cucurbitacin B was docked as the representative cucurbitacin because it showed more significant target overlap than cucurbitacin R. Diosgenin, a candidate compound with documented protective effects in experimental models of diabetic retinopathy [26,27], was included as an evidence-supported comparator. Target proteins were selected to represent distinct pathogenic axes of diabetic retinopathy while remaining traceable to the network analysis. Aldose reductase (AKR1B1) and p38α mitogen-activated protein kinase (MAPK14), which mediate the polyol pathway and inflammatory MAPK signaling, respectively, are protein targets of the candidate herbs, including Belamcandae Rhizoma, the source of tectorigenin (Table S4). VEGFA is one of the eight diabetic retinopathy-associated proteins in the disease set (Table S1); because it is a secreted ligand without a small-molecule binding pocket, its receptor tyrosine kinase VEGFR2 (KDR) was docked instead. Only proteins whose co-crystallized ligand could be redocked within 2.0 Å were retained; inducible nitric oxide synthase, although targeted by seven of the eleven candidate herbs, was excluded because its heme-coordinating native ligand could not be reproduced by redocking (RMSD 5.23 Å).

Crystal structures of aldose reductase (PDB ID 1US0; resolution 0.66 Å; co-crystallized with IDD594 and NADP^+^), VEGFR2 (PDB ID 4AG8; 1.95 Å; axitinib), and p38α (PDB ID 1A9U; 2.50 Å; SB203580) were obtained from the RCSB Protein Data Bank. Receptors were prepared by removing water molecules and heteroatoms other than the NADP^+^ cofactor of aldose reductase and by adding hydrogen atoms at pH 7.4 using PDBFixer (version 1.9), and were converted to PDBQT format with Meeko (version 0.5.0). The structures of tectorigenin (PubChem CID 5281811), cucurbitacin B (CID 5281316), and diosgenin (CID 99474) were retrieved from PubChem, their molecular formulas were verified with RDKit (version 2023.09.1), and the ligands were prepared for docking with Meeko. Molecular docking was performed using AutoDock Vina [42] (version 1.2.5) with a 20 Å cubic search space centered on the centroid of the co-crystallized ligand, the default exhaustiveness of 8, and a random seed of 42; the top-ranked pose was retained for each ligand. Docking reliability was confirmed by redocking each co-crystallized ligand, which reproduced the crystallographic pose with heavy-atom RMSDs of 0.55 Å (aldose reductase), 0.45 Å (VEGFR2), and 0.42 Å (p38α), below the 2.0 Å acceptance threshold. Docking scores were normalized by ligand size using ligand efficiency (LE; the absolute docking score divided by the number of heavy atoms) and expressed relative to that of the corresponding co-crystallized ligand (LE ratio). Docking scores were used for relative ranking of binding poses and compounds and were not interpreted as binding free energies. Docking scores of the co-crystallized ligands and all docking results are provided in Table S6.

The top-ranked docking poses of tectorigenin with VEGFR2 and aldose reductase, together with the complexes of the co-crystallized ligands axitinib (VEGFR2) and IDD594 (aldose reductase) as reference systems, were subjected to three independent 50-ns molecular dynamics (MD) simulations using OpenMM (version 8.4) [43]. Simulations used standard OpenMM settings: proteins were parameterized with the AMBER14 force field and ligands with GAFF2 (AM1-BCC partial charges), and each complex was solvated in a cubic box of TIP3P-FB water with 1.0-nm padding and 0.15 M NaCl. The NADP^+^ cofactor was retained for docking but was not included in the aldose reductase simulations. After energy minimization and 0.5 ns of equilibration, production simulations were performed at 300 K and 1 atm using a Langevin middle integrator (friction coefficient 1 ps^−1^) and a Monte Carlo barostat, with a 2-fs time step, constraints on bonds involving hydrogen, hydrogen mass repartitioning (1.5 amu), particle-mesh Ewald electrostatics, and a 1.0-nm nonbonded cutoff; the three replicates were initiated with different random velocity seeds. After periodic-boundary imaging and alignment on the protein, complex stability was assessed using the ligand heavy-atom RMSD averaged over the second half (25–50 ns) of each replicate and protein–ligand contact frequencies (Table S6).

Physicochemical properties, pharmacokinetic parameters, and drug-likeness of the three docked compounds were predicted using the SwissADME web server [44] (accessed on 30 September 2026) (Table S7).

## 5. Conclusions

In this study, we combined a multiscale interactome with a biased random-walk algorithm to systematically nominate herbal candidates and their constituent compounds for diabetic retinopathy and to infer their putative mechanisms of action. The analysis prioritized eleven previously uninvestigated herbs and a spectrum of candidate compounds—ranging from well-documented compounds such as quercetin and resveratrol to novel candidates including tectorigenin and cucurbitacin B—which were predicted to be associated with the inflammatory, oxidative-stress, and apoptotic processes central to diabetic retinopathy, including the AGE–RAGE, TNF, and NF-κB signaling axes. By integrating herbs, compounds, targets, and biological functions within a single network model, our approach moves beyond conventional single-herb or single-compound analyses to generate testable, systems-level mechanistic hypotheses. To the best of our knowledge, this is the first study to apply a biased random walk on a multiscale network to diabetic retinopathy, and it suggests that such computational frameworks can help prioritize candidates for diseases that are difficult to interrogate experimentally. However, no herb or compound was experimentally shown to prevent or treat diabetic retinopathy in this study. Accordingly, the present findings should be regarded as hypotheses that require experimental and clinical validation before any therapeutic application can be considered.

## Figures and Tables

**Table 1:** Top-ranked herbs by correlation with the diabetic retinopathy diffusion profile.

Herb Name (Latin)	Botanical Origin (Part Used)	Correlation Score	Overlap (*p*-Value #)	q-Value	Enrichment	Reported Evidence (PMID)
Pini Koraiensis Semen	*Pinus koraiensis* Siebold & Zucc., Pinaceae (seed)	0.0263	3/50 (3.09 × 10^−5^)	8.97 × 10^−4^	4.51	–
Benincasae Exocarpium	*Benincasa cerifera* Savi [*Benincasa hispida* (Thunb.) Cogn.], Cucurbitaceae (exocarp)	0.0183	2/14 (1.44 × 10^−4^)	3.36 × 10^−3^	3.84	–
Veratri Rhizoma Et Radix	*Veratrum nigrum* L. var. *ussuriense* Loes. f. or *Veratrum oxysepalum* Turcz., Melanthiaceae (rhizome and root)	0.0162	5/50 (1.93 × 10^−9^)	6.73 × 10^−7^	8.71	–
Maydis Stigma	*Zea mays* L., Poaceae (style and stigma)	0.0118	3/50 (3.09 × 10^−5^)	8.97 × 10^−4^	4.51	–
Pyrrosiae Folium	*Pyrrosia lingua* (Thunb.) Farw., *Pyrrosia petiolosa* (Christ) Ching, or *Pyrrosia tricuspis* Tagawa, Polypodiaceae (leaf)	0.0116	2/25 (4.72 × 10^−4^)	9.59 × 10^−3^	3.33	–
Tribuli Fructus	*Tribulus terrestris* L., Zygophyllaceae (ripe fruit)	0.0114	3/45 (2.24 × 10^−5^)	8.97 × 10^−4^	4.65	–
Sparganii Rhizoma	*Sparganium stoloniferum* (Buch.-Ham. ex Graebn.) Buch.-Ham. ex Juz., Typhaceae (tuber)	0.0112	3/41 (1.69 × 10^−5^)	8.97 × 10^−4^	4.77	–
Smilacis Rhizoma	*Smilax china* L. or *Smilax glabra* Roxb., Smilacaceae (rhizome)	0.0108	3/50 (3.09 × 10^−5^)	8.97 × 10^−4^	4.51	–
Belamcandae Rhizoma	*Belamcanda chinensis* (L.) DC. [*Iris domestica* (L.) Goldblatt & Mabb.], Iridaceae (rhizome)	0.0108	3/50 (3.09 × 10^−5^)	8.97 × 10^−4^	4.51	–
Benincasae Semen	*Benincasa cerifera* Savi [*Benincasa hispida* (Thunb.) Cogn.], Cucurbitaceae (seed)	0.0108	3/50 (3.09 × 10^−5^)	8.97 × 10^−4^	4.51	–
Melonis Pedicellus	*Cucumis melo* L., Cucurbitaceae (pedicel)	0.0108	3/50 (3.09 × 10^−5^)	8.97 × 10^−4^	4.51	–

The # symbol next to the *p*-value denotes values obtained from the hypergeometric test, used to assess the significance of overlap between datasets. None of the eleven top-ranked herbs has yet been directly investigated for diabetic retinopathy. Botanical origins and medicinal parts are given according to the Korean Pharmacopoeia or the Korean Herbal Pharmacopoeia; currently accepted names are shown in brackets where they differ, and family names follow current classification. q-Values were calculated using the Benjamini–Hochberg false discovery rate procedure across all 349 herbs tested.

**Table 2:** KEGG signaling pathway enrichment analysis of the core protein targets.

KEGG Pathway	Overlap	Adjusted *p*-Value	Combined Score
Pathways of neurodegeneration	15/475	9.43 × 10^−13^	744.0
Lipid and atherosclerosis	10/215	3.01 × 10^−10^	744.6
IL-17 signaling pathway	7/94	1.01 × 10^−8^	948.9
AGE–RAGE signaling pathway in diabetic complications	7/100	1.37 × 10^−8^	868.7
C-type lectin receptor signaling pathway	7/104	1.53 × 10^−8^	821.4
TNF signaling pathway	7/112	2.39 × 10^−8^	738.8

**Table 3:** Prioritized candidate compounds from the 11 candidate herbs lacking herb-level evidence and their association with diabetic retinopathy.

Herb Name (Latin)	Compound (PubChem CID)	Correlation Score	Overlap (*p*-Value #)	q-Value	Evidence (PMID)
Pini Koraiensis Semen					
Oleic acid	445639	0.0176	4/127 (3.52 × 10^−6^)	4.22 × 10^−5^	39617901
Quercetin	5280343	0.0150	5/424 (1.69 × 10^−5^)	1.01 × 10^−4^	33318415; 33469482
Stearic acid *	5281	0.0108	1/24 (0.023)	0.092	–
Benincasae Exocarpium					
β-sitosterol *	222284	0.0243	2/11 (4.47 × 10^−5^)	1.34 × 10^−4^	–
Veratri Rhizoma Et Radix					
β-sitosterol *	222284	0.0243	2/11 (4.47 × 10^−5^)	1.79 × 10^−4^	–
Resveratrol	445154	0.0170	6/326 (9.29 × 10^−8^)	7.43 × 10^−7^	23406761; 21914146; 30503749; 41606717
Diosgenin	99474	0.0113	2/25 (2.42 × 10^−4^)	6.46 × 10^−4^	36881067; 36444632
Stearic acid *	5281	0.0108	1/24 (0.023)	0.046	–
Maydis Stigma					
β-sitosterol *	222284	0.0243	2/11 (4.47 × 10^−5^)	2.46 × 10^−4^	–
Oleic acid	445639	0.0176	4/127 (3.52 × 10^−6^)	3.87 × 10^−5^	39617901
Palmitic acid	985	0.0070	2/243 (0.0213)	0.078	37566749; 41247128
Piperine	638024	0.0054	1/49 (0.0464)	0.128	34012879
Pyrrosiae Folium					
β-sitosterol *	222284	0.0243	2/11 (4.47 × 10^−5^)	1.34 × 10^−4^	–
Tribuli Fructus					
β-sitosterol *	222284	0.0243	2/11 (4.47 × 10^−5^)	1.79 × 10^−4^	–
Dioscin	119245	0.0089	1/20 (0.0192)	0.038	35153764
Sparganii Rhizoma					
Hydroxytyrosol	82755	0.0186	3/21 (7.80 × 10^−7^)	3.12 × 10^−6^	29278909
Smilacis Rhizoma					
Resveratrol	445154	0.0170	6/326 (9.29 × 10^−8^)	2.79 × 10^−7^	23406761; 21914146; 30503749; 41606717
Belamcandae Rhizoma					
Resveratrol	445154	0.0170	6/326 (9.29 × 10^−8^)	5.58 × 10^−7^	23406761; 21914146; 30503749; 41606717
Tectorigenin *	5281811	0.0097	1/15 (0.0144)	0.043	–
Benincasae Semen					
β-sitosterol *	222284	0.0243	2/11 (4.47 × 10^−5^)	1.12 × 10^−4^	–
Quercetin	5280343	0.0150	5/424 (1.69 × 10^−5^)	8.45 × 10^−5^	33469482; 33318415
Rutin	5280805	0.0085	2/45 (7.91 × 10^−4^)	1.32 × 10^−3^	31456155
Melonis Pedicellus					
Cucurbitacin R *	180535	0.0192	1/6 (0.0058)	0.021	–
Oleic acid	445639	0.0176	4/127 (3.52 × 10^−6^)	3.87 × 10^−5^	39617901
Cucurbitacin B *	5281316	0.0116	2/25 (2.42 × 10^−4^)	1.33 × 10^−3^	–
Stearic acid *	5281	0.0108	1/24 (0.023)	0.042	–
Palmitic acid	985	0.0070	2/243 (0.0213)	0.042	37566749; 41247128
Luteolin	5280445	0.0050	2/164 (0.0101)	0.028	34143751

The # symbol denotes *p*-values calculated via the hypergeometric test to assess the significance of dataset overlap. An asterisk (*) marks candidate compounds with strong associations to diabetic retinopathy that remain uninvestigated. q-Values were calculated using the Benjamini–Hochberg false discovery rate procedure among the constituent compounds of each herb; compounds with q ≥ 0.05 (stearic acid in Pini Koraiensis Semen and palmitic acid and piperine in Maydis Stigma) should be regarded as exploratory candidates. Table S3 additionally reports q-values corrected across all 946 compounds tested.

## Data Availability

The disease protein set, complete herb and compound rankings, herb–target lists, and key analysis parameters are provided in the Supplementary Materials (Tables S1–S7). The public databases used in this study are cited in the Section 4. Docking input structures, parameter files, and MD analysis scripts are available from the corresponding author upon reasonable request.

## Supplementary Materials

The following supporting information can be downloaded at: https://www.mdpi.com/article/10.3390/ijms27209066/s1.
